# Polar Body Biopsy Helps in Reducing the Total Pregnancy Loss Rates in Intracytoplasmic Sperm Injection Cycles

**DOI:** 10.7759/cureus.82160

**Published:** 2025-04-13

**Authors:** Ensar Hajder, Simon Eickhoff, Andreas Winter, Nino Jangulashvili, Elmira Hajder, Cornelius Doehmen, Ezz Al Din Alazzeh

**Affiliations:** 1 Reproductive Medicine, MVZ Amedes Fertility, Trier, DEU; 2 Institute of Neuroscience and Medicine, Brain and Behaviour (INM-7), Research Center Jülich, Jülich, DEU; 3 Institute of Systems Neuroscience and Institute of Clinical Neuroscience and Medical Psychology, Heinrich Heine University, Düsseldorf, DEU; 4 Reproductive Medicine, Woman and Health, Wien, AUT; 5 Reproductive Medicine, Christliches Klinikum Unna, Unna, DEU; 6 Reproductive Medicine, PZU Dr. Hajder, Tuzla, BIH; 7 Reproductive Medicine, Kinderwunschzentrum Niederrhein, Moenchengladbach, DEU; 8 Reproductive Medicine, Embryology, Kinderwunschzentrum Niederrhein, Moenchengladbach, DEU

**Keywords:** assisted reproductive technology (art), blastocyst, blastocyst biopsy, intracytoplasmic sperm injection (icsi), in vitro fertilization (ivf), live birth rate, miscarriage, polar body biopsy, preimplantation genetic diagnosis (pgd), single embryo transfer (set)

## Abstract

Objective: To evaluate the effect of polar body biopsy (PBB) on pregnancy and pregnancy loss outcomes in intracytoplasmic sperm injection (ICSI) cycles.

Methodology: This is a retrospective case-control study that analyzed 147 ICSI embryo transfer (ET) cycles. The study included 82 subfertile patients (31 patients with ICSI and PBB therapy = case group; 51 patients with ICSI without the PBB therapy = control group). We conducted a statistical analysis of all pregnancies and births resulting from fresh and thawed ICSI cycles, with and without the use of PBB after a single embryo transfer (SET) of a blastocyst (BL). Our main outcome measures were the pregnancy and pregnancy outcome rates after ICSI with and without the PBB.

Result: The implantation rate in the ICSI with PBB group was lower than in the ICSI without PBB group, but there were no significant differences (11 = 17.50% vs. 21 = 25.00%, RR = 0.63, 95% CI: 0.28-1.44, p = 0.37). The yolk sac detection rate in the ICSI with PBB group was lower than in the ICSI without PBB group, but there were no significant differences (8 = 12.70% vs. 16 = 19.00%, RR = 0.62, 95% CI: 0.25-1.55, p = 0.65). The fetal heartbeat rate in the ICSI with PBB group was lower than in the ICSI without PBB group, but there were no significant differences (7 = 11.10% vs. 12 = 14.30%, RR = 0.75, 95% CI: 0.28-2.03, p = 0.75). The live birth rate in the ICSI with PBB group was higher than in the ICSI without PBB group, but there were no significant differences (5 = 7.90% vs. 5 = 6.00%, RR = 1.36, 95% CI: 0.37-4.92, p = 0.88). The total pregnancy loss rate was significantly lower in the ICSI with PBB group than in the ICSI without PBB group (6 = 9.50% vs. 19 = 22.60%, RR = 0.36, 95% CI: 0.14-0.96, p = 0.04).

Conclusion: A bigger patient sample is needed for further evaluation, but based on our findings, we recommend the PBB in the cases of apparent or suspected genetic, maternal diseases and/or aneuploidies and for improving general ICSI outcomes, through the reduction of pregnancy loss rates. This information can support reproductive professionals and embryologists who are looking to invest in new solutions for their centers and labs.

## Introduction

Selecting the best possible embryo for embryo transfer (ET) is crucial for the success of assisted reproduction techniques (ART). Prolonged culture to the blastocyst (BL) stage helps the embryologist and the clinician in the selection of the most viable embryos, as BL-ET results in better pregnancy outcomes compared to cleavage stage ET [1,2]. A study indicates that 90% of embryonic chromosome abnormalities are of maternal origin [3]. More than 50% of the embryos in women of higher reproductive age are aneuploid [4]. Over the years, several valid therapies for embryo evaluation before the ET have been developed, one of which is the polar body biopsy (PBB).

Polar bodies (PB) are by-products of the meiosis of the oocyte and have no relevant function for the oocyte or the fertilization of the oocyte. The first and/or second PB can be used for preimplantation genetic diagnosis (PGD) [5,6].

The PBB is a well-known PGD method. The analysis of the first/second PB allows for genetic testing for monogenetic diseases and structural/numeric chromosome aberrations. The primary use for the PBB is the detection of maternally inherited translocations in oocytes and chromosomal aneuploidies. This can help prevent pregnancies that lead to the birth of children with (severe) genetic disorders [5,6]. The PBB, at least hypothetically, serves the secondary purpose of improving the pregnancy rates and reducing miscarriage rates in ART.

Studies on preimplantation genetic testing for aneuploidies (PGT-A) that used fluorescence in situ hybridization (FISH) failed to demonstrate improved live birth rates (LBR). New techniques, such as the array-based comprehensive genome hybridisation (aCGH) or next-generation sequencing (NGS), show better analysis precision and better results [7].

The PBB still has restrictions when compared to the trophectoderm biopsy, because it only analyses the maternal genes, whereas the trophectoderm biopsy analyses both the maternal and the paternal genes [8]. In regard to the German law situation, the PBB, when compared to the Germany-forbidden blastomere biopsy, is allowed according to the German Embryo Protection Act. The PGD is allowed in Germany only for certain indications and after the positive vote from the local Ethics Committee [6].

To our knowledge, this is one of the newest studies to critically evaluate the clinical importance of the PBB. Our aim was to provide new insights and to question the relevance of this method, since the PBB is expensive and not available in most of the institutions for assisted reproduction.

## Materials and methods

Study group

This is a retrospective, case-control study. We included 82 patient couples treated at a reproductive clinic in Germany from 13.08.2018 to 01.06.2023. Thirty-one patients had a previous intracytoplasmic sperm injection (ICSI) with PBB (case group), and 51 had a previous ICSI without the PBB (control group). Initially, 49 patients had ICSI with PBB, but 18 were then excluded due to results showing an aneuploidy after the PBB. In this study, we only analyzed ICSI BL-single embryo transfer (SET) cycles. For the control group, we included patients who had the same subfertility problems in the same timeline as the case group. The PBB indications are listed in Table 1. 

**Table 1:** Polar body biopsy (PBB) indications PBB, polar body biopsy; repeated implantation failure – no general definition: usually embryo transfer of 3 embryos of good quality with failure to concieve; habitual abortion – definition in Germany: 3 or more pregnancy losses before 20 weeks of gestation; * most common, used in this study for the subgroup definition

Advanced maternal age (usually 40 +)*
Maternal translocation or other known or suspected genetic disease(s)
Repeated implantation failure (RIF)*
(Multiple) miscarriages/habitual abortion*
Multiple IVF or ICSI cycles with subpar results (e.g. low fertilization rates)

There were 147 analyzed ET in total, 63 in the case group and 84 in the control group. A total of 104 of the 147 ET were from fresh embryo cycles (fresh-ET) and 43 from cryo-thawed cycles (KET). In the PBB group, 63 cycles in total were analyzed: 42 ET were from fresh-ET and 21 from KET cycles. In the control group, 84 total cycles were analyzed: 62 ET were from fresh-ET and 22 from KET cycles. All patients included in this study were matched to all inclusion criteria. The patient history included information on relevant operations, the presence of genetic/immunological/haematological conditions, and acute and chronic infections. The most important parameters are listed in Table 2.

**Table 2:** Clinical parameters in patients with ICSI-PBB and with ICSI without PBB Parameters are expressed as median with interquartile range (IQR) or as numbers in percent. ICSI, intracytoplasmic sperm injection; PBB, polar body biopsy; AMH, anti-Mullerian hormone; RIF, repeated implantation failure; SET, single embryo transfer; ET, embryo transfer; BMI, body mass index; NA, not applicable; NS, no significance; *chi-squared test, p<0.05; **Mann-Whitney test, p<0.05.

Parameter	ICSI with PBB	ICSI without PBB	p-value
No. of patients (n)	31	51	NA
Age in years (median with IQR)	39.00 (29.00-45.00)	37.00 (30.00-43.00)	NS**
AMH (ng/mL) (median with IQR)	1.51 (0.74-2.56)	2.02 (0.59-3.01)	NS**
≥ 40 years of age, n (%)	12/31 (38.70%)	14/51 (27.50%)	NS*
Miscarriages (2+), n (%)	13/31 (41.90%)	20/51 (39.20%)	NS*
RIF, n (%)	6/31 (19.40%)	17/51 (33.30%)	NS*
BMI (kg/m^2^) (median with IQR)	27.00 (21.35-36.55)	29.00 (22.47-37.50)	NS**
ICSI-SET, n (%)	63/63 (100%)	84/84 (100%)	NS*
ET Nr. per patient	63/31 = 2.03 (203%)	84/51 = 1.65 (165%)	NS*
ET, n (%)	63/147 (42.90%)	84/147 (57.10%)	NS*
Fresh ET, n (%)	42/63 (66.70%)	62/84 (73.80%)	NS*
Cryo-thaw ET, n (%)	21/63 (33.30%)	22/84 (26.20%)	NS*

Included in this study were patients over 40 years of age, those with a history of two or more miscarriages, and those with repeated implantation failure (RIF). We only included ICSI cycles, where fresh sperm was collected in a homologous system and used for the procedure. We excluded patients who had a double-embryo transfer (DET) or a transfer with an embryo other than a BL. In our study, we excluded cycles with aneuploid embryos, as well as patients under 18 years and over 45 years of age.

The focus of the study was the evaluation of the pregnancy outcomes after ICSI with PBB (case group), compared to ICSI without PBB (control group), given that a better understanding of these outcomes helps in deciding if the PBB as an ART add-on is worth conducting.

ICSI procedure

All 82 female patients underwent controlled ovarian stimulation with a gonadotropin-releasing hormone agonist or a gonadotropin-releasing hormone antagonist. Controlled, subcutaneous ovarian stimulation was performed. The dosage of the stimulants was administered according to the individual's weight, age, and ovarian reserve, the latter one estimated through sonography (antral follicle count, AFC) and anti-Mullerian hormone measurement (AMH).

The monitoring of the follicular development was performed with transvaginal ultrasound. The ovulation was triggered after three or more follicles reached 17 mm in size, and the oocyte pick-up took place approximately 36 hours after the ovulation induction in short sedation. An experienced gynecologist performed the oocyte pick-up with transvaginal ultrasound guidance. If there was a given indication for ICSI with/without PBB, the physician discussed the procedure in an informed consent with the patients.

**Figure 1:**
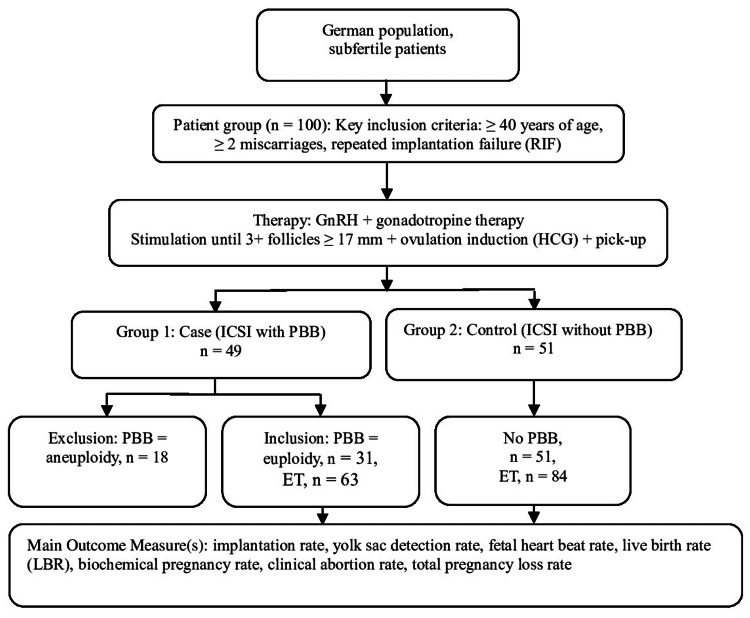
Study algorithm

The study algorithm is presented in Figure 1.

PBB procedure

**Figure 2:**
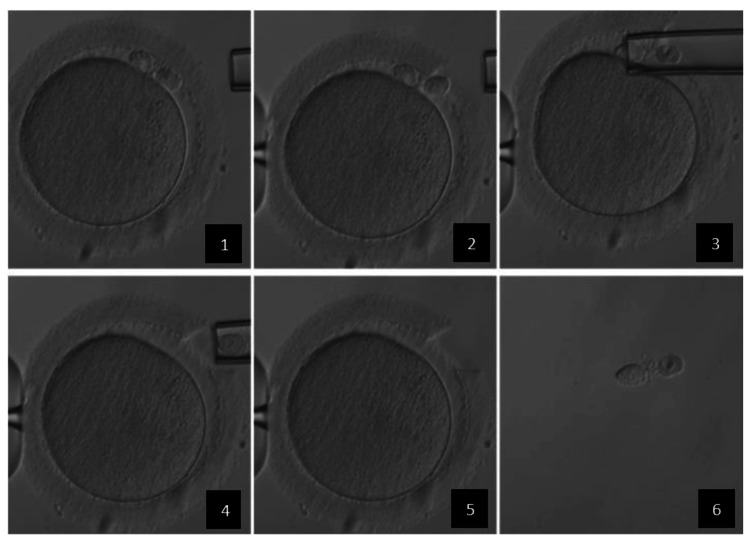
Polar body biopsy (PBB) procedure 1 – Fertilized oocyte 10 hours after ICSI with two polar bodies (PB) at 1 o'clock in the perivitelline space, 2 – assisted hatching of zona pellucida with a RI Saturn 5 laser system (Cooper surgical), 3/4 – withdrawal of the two PB with a pipelle, 5 – no visible damage to the embryo structure after the withdrawal of the PB, 6 – two visible PB after the withdrawal in the medium.

Briefly, for the ICSI, the cumulus-oocyte complexes (COCs) were isolated from the follicular fluid and then rinsed, cultured, and incubated. On the day of oocyte pick up, the total motile count was routinely determined after sperm processing by gradient density centrifugation. After that, we proceeded with the ICSI fertilization. Fertilization was identified by the presence of two pronuclei. One day before PBB, the biopsy dish has been prepared. Then, the embryologist hatched the zona pellucida and carefully performed the withdrawal with a pipette and the biopsy of the PB with a biopsy needle. The two PBs were then placed in separate drops on the dish. For fresh-ET PBB cycles, the tubes were picked up and transported in refrigerated medium by courier on dry ice. For KET PBB cycles, the biopsy was initially stored at -20°C (Figure 2).

The process was repeated until all pronuclear stages had undergone the PBB. We conducted a aCGH analysis of all PB in all cases. For the evaluation of all embryos in our study, we used a modified Gardner and Schoolcraft scoring system. No hatching or hatched BL were considered in our study. The Fresh-ET and KET were conducted according to the standard operating procedures of the clinic. The culture conditions and the medium volume used were the same for FET and KET cycles.

The biochemical pregnancy was investigated 14 days after ET (ET + 14) by a blood β-hCG test in our center, and an ongoing pregnancy was confirmed by a fetal heartbeat during an ultrasound at approximately six weeks of gestation (ET + 30/31). The patients or the treating gynecologist gave us input in regards to the miscarriages after these occurred or the birth approximately four to eight weeks after the birth via standardized questionnaires.

Study outcome definitions

The implantation rate was defined by the number of positive blood HCG tests 14 days after ET (ET + 14 days), divided by the ET number: Implantation rate = number of positive HCG tests / ET number. The yolk sac detection rate was defined by the number of yolk sacs observed via ultrasonography, divided by the ET number (ET + 30/31 days): Yolk sac detection rate = number of pregnancies with observed yolk sac / ET number. The clinical pregnancy rate (CPR) with fetal heartbeat was defined by the number of clinical pregnancies with an observed yolk sac and a positive heartbeat 30/31 days after ET (ET + 30-31 days), divided by the ET number: Clinical pregnancy rate = pregnancy number with positive heartbeat / ET number. The live birth rate (LBR) was defined as the number of births after the ET, divided by the ET number: LBR = number of births after ET / ET number. Biochemical pregnancy was defined as the absence of an observed pregnancy in the ultrasound examination, despite a positive urine or blood hCG pregnancy test. Biochemical pregnancy loss rate was defined by the number of pregnancies with a positive HCG test, with the absence of a visible pregnancy via ultrasonography, divided by the ET number: Biochemical pregnancy loss rate = Implantation rate - CPR / ET number. The clinical miscarriage rate was defined by the loss of pregnancy after an observed positive fetal heartbeat prior to the miscarriage, divided by the ET number: Clinical miscarriage rate = CPR with fetal heartbeat - LBR / ET number. The total pregnancy loss rate was calculated as follows: Total pregnancy loss rate = biochemical pregnancy loss rate + clinical miscarriage rate / ET number.

Statistical analysis

The statistical analysis was performed with Statistical Product and Service Solutions (SPSS, version 27.0; IBM SPSS Statistics for Windows, Armonk, NY). Numeric variables are expressed as median with IQR, categorical variables are expressed as numbers (n) in percentages (%). Data were analyzed using the Mann-Whitney U test for comparisons of nonparametric variables. Pearson's chi-squared test was used for categorical variables (sample size n>5), and the two-sided Fisher's exact test was used for categorical variables (sample size n<5). The strength of the association between categorical variables was measured using the odds ratio (RR) and 95% confidence intervals (CI). A p-value of ≤ 0.05 was considered statistically significant.

Ethics declaration

The study was approved by the local ethics committee in Duesseldorf (Number 134/2024). The authors declare that they have no competing interest.

## Results

The aim of this study was to show that the PBB, in combination with the ICSI treatment for infertility, has no significant positive impact on improving the pregnancy rates in ART, when compared to ICSI without the PBB. On the other hand, we wanted to demonstrate the positive effect of the PBB on the miscarriage rates. We analyzed the results of 147 ET in total.

In our study, the PBB, as an add-on, had no significant effect on the pregnancy rates, but a significant effect on the miscarriage rates.

The implantation rate in the ICSI with the PBB group was lower than in the ICSI without the PBB group, but there were no significant differences (11 = 17.50% vs. 21 = 25.00%, RR = 0.63, 95% CI: 0.28-1.44, p = 0.37). The yolk sac detection rate in the ICSI with the PBB group was lower than in the ICSI without the PBB group, but there were no significant differences (8 = 12.70% vs. 16 = 19.00%, RR = 0.62, 95% CI: 0.25-1.55, p = 0.65). The fetal heartbeat rate in the ICSI with the PBB group was lower than in the ICSI without the PBB group, but there were no significant differences (7 = 11.10% vs. 12 = 14.30%, RR = 0.75, 95% CI: 0.28-2.03, p = 0.75). The live birth rate in the ICSI with the PBB group was higher than in the ICSI without the PBB group, but there were no significant differences (5 = 7.90% vs. 5 = 6.00%, RR = 1.36, 95% CI: 0.37-4.92, p = 0.88) (Table 3). 

**Table 3:** Pregnancy outcomes in patients with ICSI-PBB and with ICSI without the PBB Parameters are expressed as numbers in percent. ICSI, intracytoplasmic sperm injection; PBB, polar body biopsy; chi-squared test, p<0.05; CI, confidence interval; df, degrees of freedom

Parameters n, %	ICSI with PBB, n = 63 ET	ICSI without PBB, n = 84 ET	Rate Ratio (95% CI)	df	p-value
Implantation rate n, %	11 (17.50%)	21 (25.00%)	0.635 (0.28-1.44)	1	0.37
Yolk sac detection rate n, %	8 (12.70%)	16 (19.00%)	0.62 (0.25-1.55)	1	0.65
Fetal heart beat detection rate n, %	7 (11.10%)	12 (14.30%)	0.75 (0.28-2.03)	1	0.75
Live birth rate n, %	5 (7.90%)	5 (6.00%)	1.36 (0.37-4.92)	1	0.88

The biochemical pregnancy rate in the ICSI with the PBB group was lower than in the ICSI without the PBB group, but there were no significant differences (4 = 6.30% vs. 10 = 11.90%, RR = 0.50, 95% CI: 0.15-1.67, p = 0.39). The clinical miscarriage rate in the ICSI with the PBB group was lower than in the ICSI without the PBB group, but there were no significant differences (2 = 3.20% vs. 9 = 10.70%, RR = 0.27, 95% CI: 0.06-1.31, p = 0.12). The total pregnancy loss rate was significantly lower in the ICSI with the PBB group than in the ICSI without the PBB group (6 = 9.50% vs. 19 = 22.60%, RR = 0.36, 95% CI: 0.14-0.96, p = 0.04) (Table 4).

**Table 4:** Pregnancy loss outcomes in patients with ICSI-PBB and with ICSI without the PBB Parameters are expressed as numbers in percent ICSI, intracytoplasmic sperm injection; PBB, polar body biopsy; **chi-squared test (variant: *Fisher's exact test), p<0.05; CI, confidence interval; df, degrees of freedom

Parameters n, %	ICSI with PBB, n = 63 ET	ICSI without PBB, n = 84 ET	Rate Ratio (95% CI)	df	p-value
Biochemical pregnancy rate n, %	4 (6.30%)	10 (11.90%)	0.50 (0.15-1.67)	1	0.39*
Clinical miscarriage rate n, %	2 (3.20%)	9 (10.70%)	0.27 (0.06-1.31)	1	0.12*
Total pregnancy loss rate n, %	6 (9.50%)	19 (22.60%)	0.36 (0.14-0.96)	1	0.04**

## Discussion

The aneuploidy of the gametes is one of the causes of infertility, and 90% of embryonic chromosome abnormalities are of maternal origin [3]. Over the years, valid strategies for embryo evaluation before the ET have been developed, one of which those the PBB. The primary use for the PBB is the detection of maternally inherited translocations in oocytes and chromosomal aneuploidies. With this, we can discard aneuploid and only use euploid embryos for our therapies. This helps us prevent pregnancies that lead to the birth of children with genetic disorders [5,6]. The PBB, at least hypothetically, serves another purpose in improving the pregnancy rates and reducing the miscarriage rates in ART.

In our study, we proved that the PBB as an ICSI add-on does not improve pregnancy or live birth rates, but reduces the total pregnancy loss rates significantly. This is in concordance with the results of some studies, whereas other studies have shown different results.

Feichtinger et al. showed that the PBB group had a significantly higher LBR per embryo, when compared with the control group (26.40% vs. 14.90%) [9].

van der Ven et al. conducted a study in 2008 in regards to aneuploidy screening. The results showed a significantly higher implantation rate in the PBB group, when compared with the control group in women between 35 and 39 years of age and with at least two previous in vitro fertilization (IVF) attempts: 17.50% (44/251) vs. 11.80% (35/297), p < 0.05. The difference in the clinical pregnancy rate per transfer and the birth rate per cycle and per transfer was not significant. The results of PBD for aneuploidy screening in women aged PBD group 40 years and older showed no significant differences [6]. More research on this topic is needed.

In a study conducted by Ebner et al. in 1999, the data of 212 transferred embryos in the study group were analyzed and compared with the data of the ET of 313 embryos in the control group in regards to the outcomes implantation and pregnancy rates. The implantation and pregnancy rates were signicantly higher in the study group, when compared with the control group [10].

On the other hand, Haaf et al. conducted their research in 2009 on 607 women undergoing ICSI and PBB and on 591 women undergoing ICSI without the PBB, to compare the pregnancy rates and LBR. The pregnancy rates (17.30% vs. 51.80%) and LBR (10.90% vs. 43.00%) were significantly lower in women undergoing ICSI with the PBB, when compared to women without the PBB [11].

Griesinger et al. conducted a PBB in a series of nine German patient couples with a risk of transmitting a monogenetic disease in the year 2009: the authors demonstrated a live birth rate of 30% after the PBB [12]. The results are similar to the usual live birth rates in Germany without the PBB. The design of this small clinical series did not demonstrate the superiority of PBB when compared to controls but showed the possibilities of the PBB method. A bigger, homogeneous cohort would be needed for more significant results.

The PBB is in many cases a treatment option and not an obligatory add-on. Considering this, there should be no negative consequences of this procedure on the embryo quality and/or development. The safety of the PBB procedure was discussed a lot in the literature.

No data revealed a significant negative impact on embryo development and pregnancy or birth rates. Schenk et al. demonstrated that the PBB does not negatively impact the morphokinetics of embryo development [13]. In a study from 2014, Eldar-Geva et al. did not show a negative effect on the neonatal outcome following the PBB [14]. Kuliev et al. highlighted the safety of the procedure in their study from 2011 [15]. Strom et al. demonstrated no significant decrease in birth length, weight, or the number of small for gestational age (SGA) babies from a study dating from 2000. The authors showed no relevant negative effects of the PBB on the children born after ART in combination with the PBB [16].

In regard to the economic perspective, Neumann et al. [17] conducted an analysis of the cost per live birth and the cost of preventing a miscarriage in ART, combined with polar body biopsy and aCGH in the year 2020. The authors compared these costs with the costs of the regular IVF/ICSI treatment, without the PBB add-on, in women 36-40 years of age. ART with the PBB increased the cost per live birth by approximately 15%-285%, depending on the scenario. A further analysis revealed that the PBB would need to be associated with an absolute increase in pregnancy rates from 6% to >39%. The incremental cost to prevent a single miscarriage by the PBB was calculated to be US$ 51,146 [17]. In regard to these data, we need to be careful where we use this method, because of the high associated costs for the patients.

One of the clear indications for PBB would be the reduction of the total pregnancy loss rates in a specific cohort where pregnancy losses occurred (e.g., patients with habitual pregnancy losses).

In regards to this study, the positive aspects would be a carefully matched population, data from a center with very good procedure and outcome results and the focus on the relevance of the topic that it adresses. The limitations of this study are a relatively small, unicentric cohort, the retrospective character, and the mix of the analyzed fresh and cryo-thaw cycles.

## Conclusions

While the PBB did not significantly enhance implantation rates, fetal yolk sac detection rates, fetal heartbeat detection rates, or live birth rates in patients undergoing ICSI, it was associated with a statistically significant reduction in total pregnancy loss rates.

We still see the clear indication for the PBB in female patients with higher risk for aneuploidies and/or with known or suspected genetic problems. We have shown that the PBB should not be used explicitly for optimizing the pregnancy rates or live birth rates, but for reducing the total pregnancy loss rates in a specific cohort where pregnancy losses occurred.
